# Bis(vinylenedithio)tetrathiafulvalene analogues of BEDT-TTF

**DOI:** 10.3762/bjoc.11.46

**Published:** 2015-03-27

**Authors:** Erdal Ertas, İlknur Demirtas, Turan Ozturk

**Affiliations:** 1 TUBITAK Marmara Research Center, FI, P.O.Box 21, 41470 Gebze-Kocaeli, Turkey; 2 Istanbul Technical University, Science Faculty, Chemistry Department, Organic Chemistry, 34469 Maslak, Istanbul, Turkey; 3 TUBITAK UME, Chemistry Group Laboratories, P.O.Box 54, 41470 Gebze-Kocaeli, Turkey

**Keywords:** bis(ethylenedithio)tetrathiafulvalene, bis(vinylenedithio)tetrathiafulvalene, tetrathiafulvalene

## Abstract

This review aims to give an overview of the current status of our research on the synthesis of π-electron donor bis(ethylenedithio)tetrathiafulvalene (BEDT-TTF, ET) analogues prepared from 1,8-diketones via a ring forming reaction. The new synthesized π-electron donors have vinyl moieties producing extended π-electron delocalization over the substituent phenyl rings at the peripheries.

## Introduction

**Figure 1:**
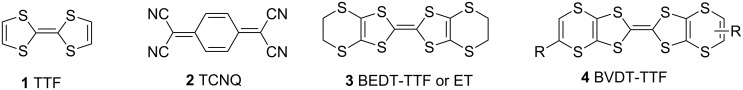
Chemical structure of the TTF analogues and TCNQ.

Tetrathiafulvalene (TTF, **1**, Figure 1) was first synthesized in 1960s by Klingsberg’s method [1]. Shortly after this, it was synthesized by other research groups and used as a donor molecule in 1970 [2]. Although, in 1972, **1** was demonstrated to be an organic material conductor in the form of its chloride salt [3]. The interest in the chemistry of **1** begun with the discovery of the salt of **1** with 7,7,8,8-tetracyanoquinodimethane (**2**, TTF-TCNQ) in 1973 [4]. Since then, studies have been focused on the syntheses of donor TTF analogues and investigations of the physical properties of their charge-transfer (CT) salts with various acceptors for applications such as electrically conductive materials, super conductive materials, magnetic substances, electrochromic materials, electroluminescent materials, etc. [5–16]. TTF-TCNQ, which is metallic under 54 K and known to be the first true one-dimensional synthetic metal, led to the production of superconducting salts based on TTF type donors containing a heteroatom such as sulfur, selenium, oxygen, etc. [17–20]. Among a large number of tetrathiafulvalene analogues, bis(ethyleneditiho)tetrathiafulvalene (BEDT-TTF, **3**), also known as ET, has been the most studied and has had the largest number of radical cation salts of its CT materials investigated at very low temperature [12,21–24].

In order to improve the properties of TTF type materials, various methods have been applied, including extension of π-conjugation through double bonds [25–30] and fused aromatic rings [31–34] and the construction of molecules having acceptor substituents [35–37]. Generally, all these modifications served to produce molecules with better conjugation and improved S···S intermolecular and C–H···anion interactions in determining the solid state properties [35–37]. Bis(vinylenedithio)tetrathiafulvalene (BVDT-TTF) **4** (R = Ph, 4-CH_3_OC_6_H_4_, 4-BrC_6_H_4_, 4-CH_3_C_6_H_4_, 4-O_2_NC_6_H_4_, 2-thienyl) is a BEDT-TTF analogue possessing π-bonds with aromatic groups on the outer rings (Figure 1) [26,38–41]. Since BEDT-TTF has two ethylene units at the both ends of the molecule, it has a non-planar structure [42]. π-Extended molecules such as **4** with a vinylene group at the end of the BEDT-TTF unit have more planar structures [41,43]. Further, a tetrathiafulvalene with a fused aromatic heterocycle was synthesized as a π-extended donor molecule [28,40]. The most notable superconductivity was observed with the radical cation salts derived from the electron-donor molecule bis(ethylenedithio)tetrathiafulvalene (BEDT-TTF) as a (BEDT-TTF)_2_Cu[N(CN)_2_]Br salt at 12.5 K (resistive onset) [24].

**Figure 2:**
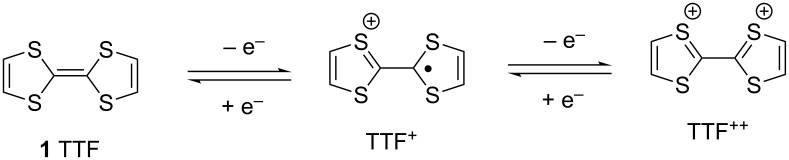
Oxidation states of TTF.

The tetrathiafulvalene (TTF) ring system is one of the most intensively studied redox-active organic molecules. It has two easily accessible oxidized states, TTF^+^ and TTF^2+^ with potentials of *E*_1_^1/2^ = +0.34 and *E*_2_^1/2^ = +0.78V, respectively, using Ag/AgCl in acetonitrile, (Figure 2) [5–6,44].

TTF analogues have been synthesized by coupling and without coupling methods [45–46]. Depending on the presence of electron-withdrawing groups on the TTF, they exhibit various oxidation potential ranges [15,26]. Recently, TTF and analogues have received widespread attention involving the development of new materials by using various anions to form different charge transfer salts. The physical and electronic properties of their solid states were investigated [13,25,47–49].

We attempt here to provide a summary of the synthesis of differently functionalized and extensively π-electron delocalized conjugated TTF core dithiin- and thiophene-fused donor molecules, obtained from 1,8-diketone ring closure reactions, and coupling reactions, published by our group.

## Review

### BVDT-TTF analogues from 1,8-diketones

**Figure 3:**
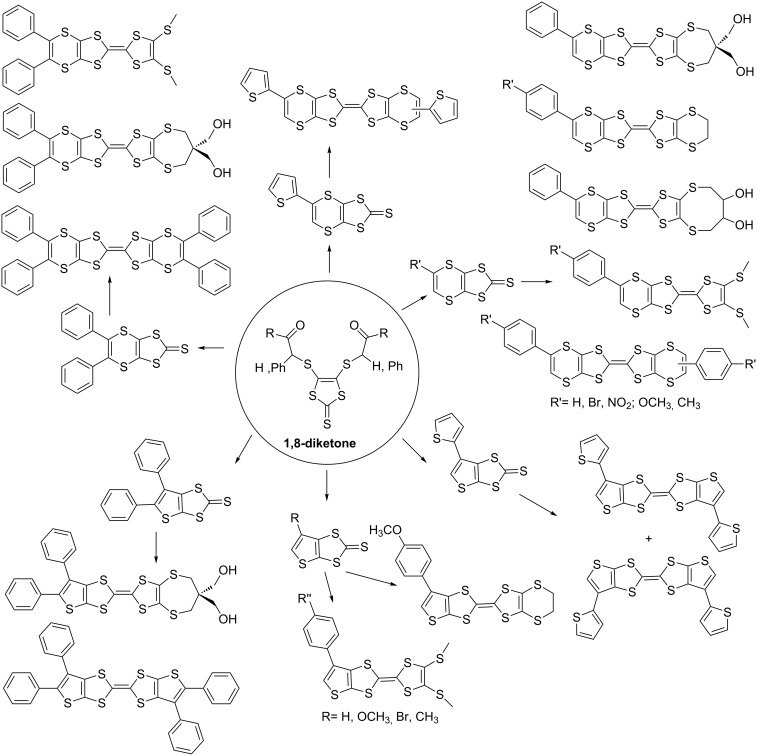
1,4-Dithiin and thiophene fused TTF analogues from 1,8-diketone.

Bis(vinylenedithio)tetrathiafulvalene (BVDT-TTF) **4** (R = Ph, 4-CH_3_OC_6_H_4_, 4-BrC_6_H_4_, 4-CH_3_C_6_H_4_, 4-O_2_NC_6_H_4_, 2-thienyl) is a fully unsaturated analogue of BEDT-TTF (ET) **3**. It possesses a vinyl moiety at the peripheries in place of the ethylene group of ET. It can also be considered as a tetrathiafulvalene analogue having fused 1,4-dithiin rings as its peripheries. The synthesis was achieved through the reaction of a 1,8-diketone with Lawesson’s reagent (LR) [50] or tetraphosphorus decasulfide (P_4_S_10_) [51] Although, in most cases, formation of 1,4-dithiins is the only result, or the major one, a thiophene formation can also take place [46]. So far, eighteen BVDT-TTF analogues have been synthesized (Figure 3).

**Scheme 1:**
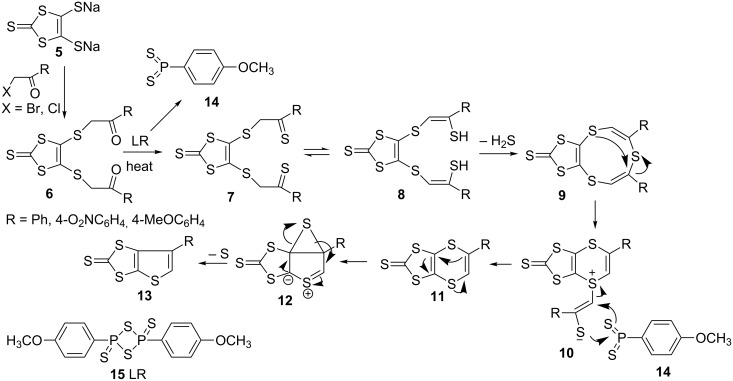
Reaction mechanism of fused 1,4-dithiin and thiophene ring systems.

In 1996, we reported a convenient method of synthesizing fused 1,4-dithiin and thiophene ring systems, possessing functional groups such as Ph 4-MeOC_6_H_4_ and 4-O_2_NC_6_H_4_ (Scheme 1) [46]. The synthesis involved treatment of the diketone **6**, produced through the reaction of the readily available dianion **5** [52] with α-haloketones, with Lawesson’s reagent **15** to obtain [1,3-dithiolo[4,5-*b*][1,4]dithiin-2-thione **11**, which is an analogue of half ET, as a major product, and the thiophene **13** as a minor product.

**Scheme 2:**
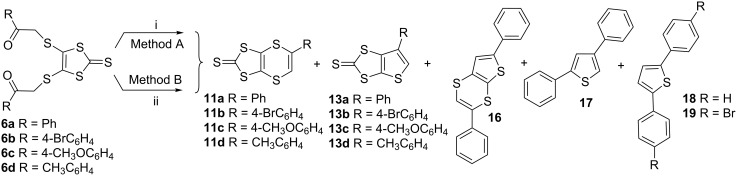
Reaction conditions (i) LR, toluene, reflux, overnight; (ii) P_4_S_10_, toluene, reflux, 3 h.

After employing different reaction conditions and an in depth study, we suggested that the reaction mechanism involves interaction of **6** with LR **15** (refluxing toluene) initially leading to the formation of enethiols **8**, a tautomer of **7**, then nine-membered ring **9**, rearrangement of which produces **10**. Lastly, the reaction of **10** with fragment **14** of LR would give **11** as a major product (Scheme 1). Rearrangement of the 1,4-dithiin unit of **11** would produce **13** as a minor product through the intermediate **12** by the loss of elemental sulfur. The reaction of a series of 1,8-diketones with LR **15** or P_4_S_10_ was further explored in 2003 [53]. With both reactants, 1,4-dithiin **11** was obtained as a major and thiophene **13** as a minor product along with the side products **16**–**19** (Scheme 2).

Depending on the electron-releasing or electron-withdrawing nature of the groups on **6**, the yields for **11** and **13**, with LR varied between 35–52% and from not detected (n.d.) to 18%, respectively, P_4_S_10_ gave yields for **11** and **13** from 5 to 49% and n.d. to 27%, respectively. Both of the reagents produced the dithiin as a major product. Compound **13d**, with a thiophene ring, was not obtained with either of the two reagents (Table 1).

**Table 1:** Ring closure methods and product yields.

Starting material	LR		P_4_S_10_	
	
	dithiin	thiophene	dithiin	thiophene

**6a**	**11a** (40%)	**13a** (17%) **16** (15%) **17** (<1%) **18** (9%)	**11a** (49%)	**13a** (n.d.) **17** (8%) **18 (**n.d.)
**6b**	**11b** (35%)	**13b** (18%)	**11b** (40%)	**13b** (2%), **19** (10%)
**6c**	**11c** (45%)	**13c** (15%)	**11c** (30%)	**13c** (27%)
**6d**	**11d** (52%)	**13d** (n.d.)	**11d** (5%)	**13d** (n.d.)

n.d.: not detected.

**Scheme 3:**
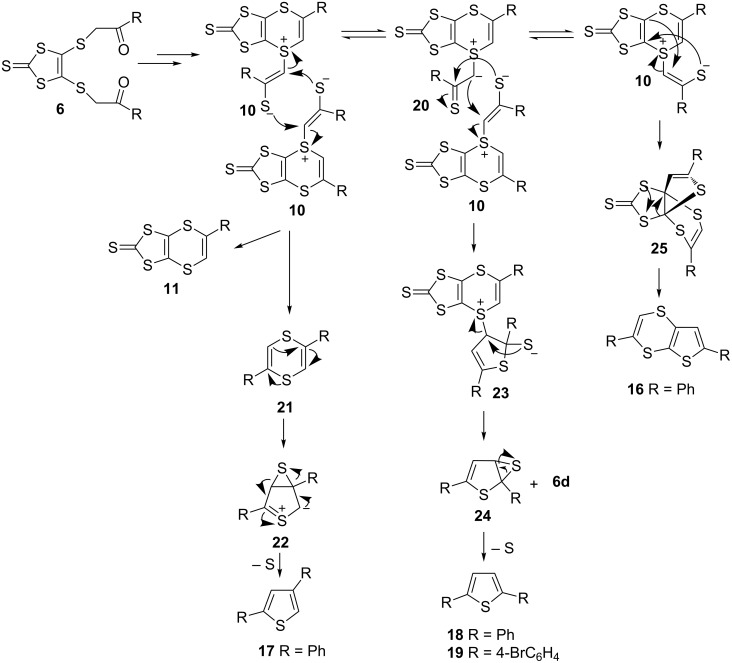
Proposed mechanism for side products.

A possible reaction mechanism for the formation of **16**–**19** was suggested to involve the intermediate **10** (Scheme 3) [46–53]. A detailed semi-empirical PM3 calculation indicated that the formation of the intermediate **10** is an endothermic process with Δ*H*_rxn_= 29.435 kcal/mol. The reaction of the intermediate **10** with itself could produce the 1,4-dithiin ring **11** and the side product thiophene **17** through the intermediates **21** and **22** by removal of elemental sulfur (Scheme 3). The other side products **18** and **19** were possibly formed from the reaction of **10** with **20**, leading to formation of **23** and **24**, rearrangement of which would then produce **18** and **19**. Moreover, rearrangement of **10** via **25** would result in the formation of **16**. The structures of the side products **16**–**19** can be taken as evidence for the proposed reaction mechanism.

**Scheme 4:**
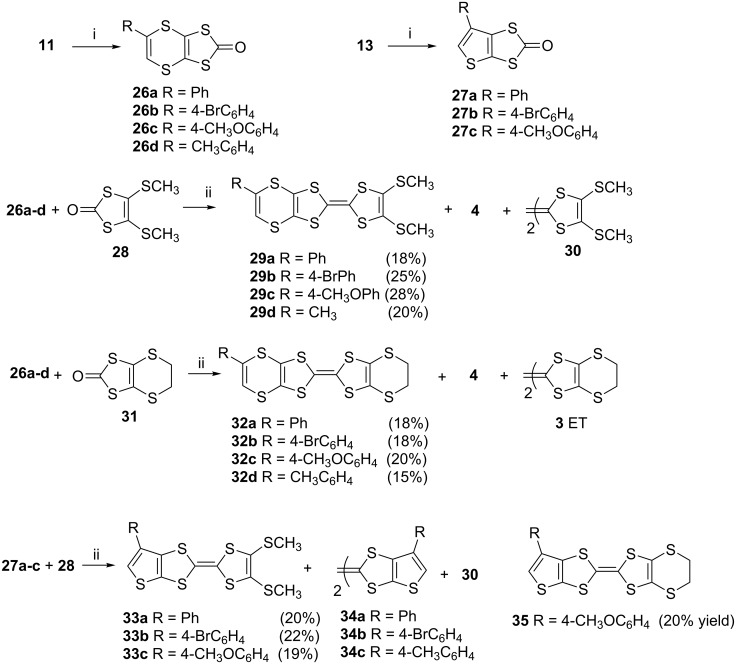
Reaction conditions (i) Hg(OAc)_2_, AcOH/CHCl_3_, rt, 1h; (ii) (EtO)_3_P, N_2_, 3 h, 110 °C.

The thione sulfur atoms of **11a**–**d** and **13a**–**c** were converted into their corresponding oxo forms **26a**–**d** and **27a**–**c**, respectively, using mercury acetate (Scheme 4) [46,53–54]. These were then subjected to cross coupling reactions. While the cross couplings of **26a**–**d** with **28** and **31** [53] led to the formation of **29a**–**d** and **32a**–**d**, respectively, along with the self coupling products **4**, **30** and **3**, coupling of **27a**–**c** with **28** gave **33a**–**c** and the self coupling products **34a**–**c** and **30**. The cross coupled product **35** from **27c** and **31** was obtained in a similar manner.

The redox properties of the donor molecules **29a**–**d**, **32a**–**d**, **33a**–**c** and ET **3** were studied by cyclic voltammetry in solution in acetonitrile, containing NaClO_4_ and dichloromethane, containing tetrabutylammonium tetrafluoroborate (TBABF_4_) (Table 2 and Table 3). Measurements were performed under a nitrogen atmosphere at room temperature using Pt as working and counter electrodes and Ag/AgCl reference electrode. The oxidation potentials of the coupled products were compared with ET **3**.

**Table 2:** Redox potential of **29** and ET **3**. in 1 mM MeCN solution, NaClO_4_ (0.1 M) vs Ag/AgCl, 100 mVs^−1^.

Donor	*E*^1^_ox_ (V)	*E*^2^_ox_ (V)	Δ*E*_ox_ (V)

**29a**	0.49	0.63	0.14
**29b**	0.50	0.63	0.13
**29c**	0.47	0.72	0.25
**29d**	0.42	0.66	0.24
**3** ET	0.50	0.77	0.27

**Table 3:** Redox potential of **32a**–**d** and **33a**–**c**. and ET **3** in 1 mM CH_2_Cl_2_ solution, TBABF_4_ (0.1 M) vs Ag/AgCl, 115 mVs^–1^.

Donor	*E*^1^_ox_ (V)	*E*^2^_ox_ (V)	Δ*E*_ox_ (V)

**32a**	0.66	0.96	0.30
**32b**	0.60	0.95	0.35
**32c**	0.68	1.00	0.32
**32d**	0.64	0.99	0.24
**33a**	0.59	0.86	0.27
**33b**	0.51	0.83	0.32
**33c**	0.62	0.94	0.32
**3** ET	0.51	0.85	0.34

The measurements indicated that as the first oxidation potential of ET was higher than the first oxidation potential of **29a**, **29c** and **29d**, the oxidation potential of **29b** was equal to that of ET **3** and the second oxidation potentials of **29a**–**d** were found to be lower than for ET. On the other hand, the first and second oxidation potentials of the donors **33a**–**c** were slightly higher than the oxidation potentials of ET. The oxidation potentials of the donor molecules **32a**–**d** were higher than the ET **3** oxidation potential.

**Scheme 5:**
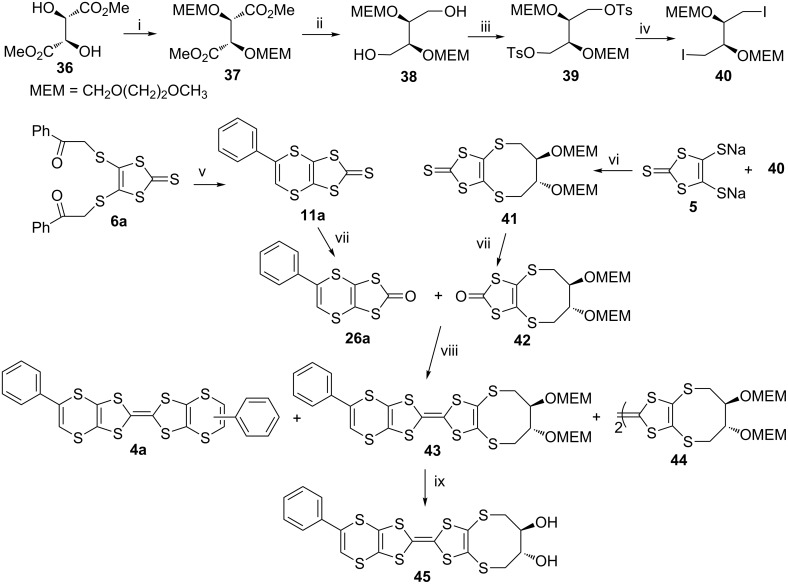
Reaction conditions (i) iPr_2_NEt, MEMCl, THF, rt, 12 h; (ii) LiAlH_4,_ dry ether, rt, 24 h; (iii) tosyl chloride, dry pyridine, 0 °C, 4 h; (iv) KI, dry acetone, N_2_, reflux, overnight; (v) dry THF, 75 °C, N_2_, 48 h; (vi) P_4_S_10_, toluene, reflux, 3 h; (vii) Hg(OAc)_2_, AcOH/CHCl_3_, rt, 2 h; (viii) neat (EtO)_3_P, N_2_, 3 h; (ix) sample in THF at 0 ^o^C , add 20% HCl, then rt., overnight.

A BEDT-TTF analogue containing phenyl-l,4-dithiin and 2,3-dihydroxybutane-l,4-dithio at the periphery, **43**, was reported as a new highly functionalized donor molecule (Scheme 5) [54].

The reaction of diketone **6a**, with LR **15** in refluxing toluene for 3 h gave the dithiin **11a**, which was converted into the oxo form **26a** with mercuric acetate in CHCl_3_/glacial acetic acid at room temperature in 2 h. Synthesis of **41** was completed in five steps, starting from dimethyl L-tartrate **36**, the hydroxy groups of which were protected by reaction with methoxyethoxymethyl chloride (MEMCl) and then the ester groups of **37** were reduced to alcohols with LiAlH_4_ to obtain the diol **38**. This was converted into **39** through tosylation of the hydroxy groups with tosyl chloride and then conversion into iodides **40** using potassium iodide. Treatment of **40** with the dianionic salt **5** in dry acetone at room temperature produced **41** [55], which was transformed into the corresponding oxo form **42** by applying the same reaction conditions used to obtain **26a**. Coupling of **26a** with **42** was performed in neat triethyl phosphite at 130 °C for 3 h under a nitrogen atmosphere, which gave a mixture of cross coupled **43** and self coupled products **4a** and **44**. In order to remove the MEM protecting group, **43** was stirred in 20% HCl at room temperature for 2 days, which yielded the ET analogue **45**, having two hydroxy groups.

**Scheme 6:**
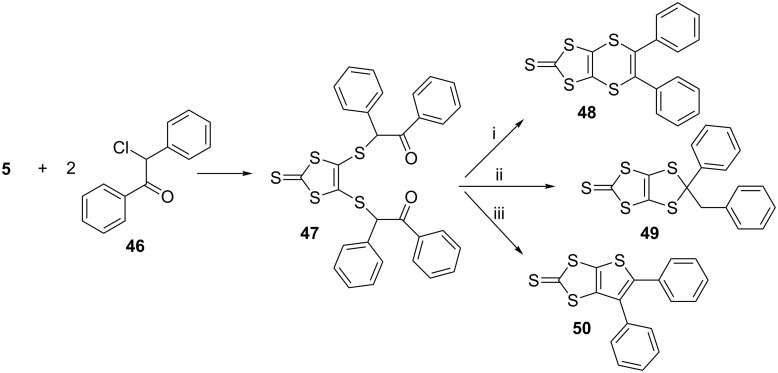
Reagents and conditions (i) P_4_S_10_, toluene, reflux, dark, 3 h; (ii) P_4_S_10_, toluene, reflux, 3 h; (iii) LR, toluene, reflux, overnight.

In 2000, syntheses of 5,6-diphenyl[1,3]dithiolo[4,5-*b*][1,4]dithiin-2-thione **48** and its coupling product **52**, which is a fully unsaturated analogue of BEDT-TTF, were achieved. The 1,8-diketone **47** was easily obtained from the reaction of the dianion **5** (1 equiv) and desyl chloride **46** (2 equiv) in dry ethanol at room temperature for 3 h in 90% yield (Scheme 6) [40].

**Scheme 7:**
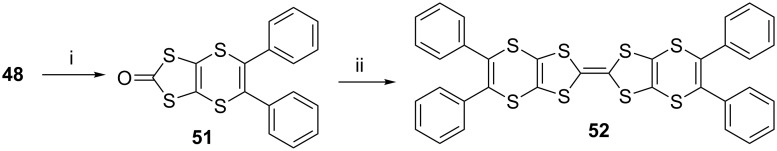
Reagents and conditions (i) Hg(OAc)_2_–AcOH, CHCl_3_, 3 h, rt; (ii) (EtO)_3_P, 110 °C, N_2_, 2 h.

The ring closure reaction of **47** was performed initially using LR, which produced only the thiophene **50**, similar to the result obtained by another research group [28]. Next, the reaction was conducted with P_4_S_10_, which gave benzylphenyldithiole **49** and the thiophene **50** in 25 and 30% yields. Considering that the reaction could take place through a radical mechanism, it was repeated in the dark using P_4_S_10_. After 3 h of reflux in toluene, the dithiin **48** was successfully obtained in 65% along with a trace of benzylphenyldithiole **49** and the thiophene **50** in 20% yields. The fully unsaturated tetraphenyl analogue **52** of ET was obtained in 90% yield by a coupling reaction of **51**, which was obtained by converting the thione group of **48** to its corresponding oxo form in 85% yield, in hot triethyl phosphite, yielding **52** in 90% yield (Scheme 7).

**Scheme 8:**
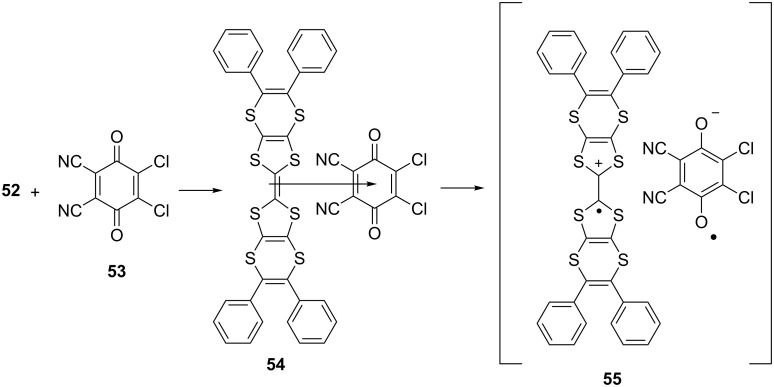
Charge transfer complex of 5,5',6,6'-tetraphenyl-2,2'-bi([1,3]dithiolo[4,5-*b*][1,4]dithiinylidene) **52** – DDQ **53**.

A charge transfer salt **54** of **52** was prepared with the acceptor 2,3-dichloro-5,6-dicyano-*p*-benzoquinone (DDQ) **53** (1:1) in dichloromethane at room temperature to investigate the optical constant and optical band gap of the complex (Scheme 8) [56]. A solution of the salt was evaporated on a quartz substrate until ≈110 nm thickness of the film was obtained. The film was annealed at 25, 55, 85, 115 and finally at 145 °C for 30 min.

Electronic transitions of the complex **54**, i.e. n–π* and π–π* transitions, led to the formation of radical ion pairs **55**. The refractive index dispersion and optical constant of the annealed film were examined for each temperature. The absorbance, refractive index, reflectance and transmittance values of the material were found to be between 0.16–0.32, 2.3–2.7, 16–20% and 46–66%, respectively at 400–800 nm wavelength range which clearly indicated that the refractive index, absorbance and reflectance of the complex decreased while transmittance increased with increased annealing temperature.

**Scheme 9:**
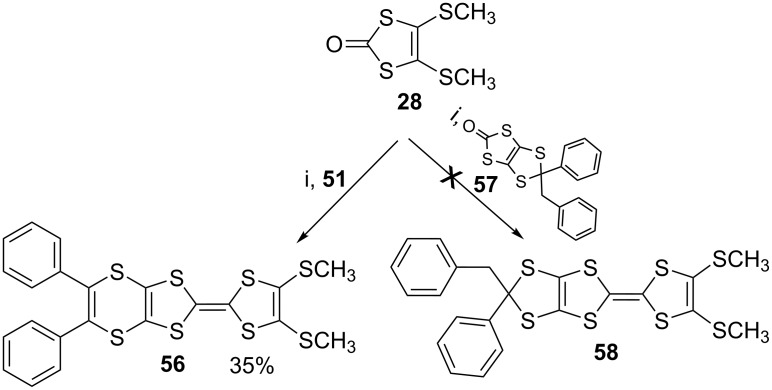
Reaction conditions (i) (EtO)_3_P, 110 °C, N_2_, 2 h.

Our easy synthesis of dithiin-containing compounds led to the production of various BEDT-TTF analogues, comprising monophenyldithiin, diphenyldithiin, diphenylthiophene and diols [40,46,53]. While coupling of **28** with **51** smoothly gave the corresponding ET analogue **56**, its reaction with **57** did not produce any result (Scheme 9) [57]. This could be due to the reaction of the benzylphenyldithiole moiety with triethyl phosphite.

**Scheme 10:**
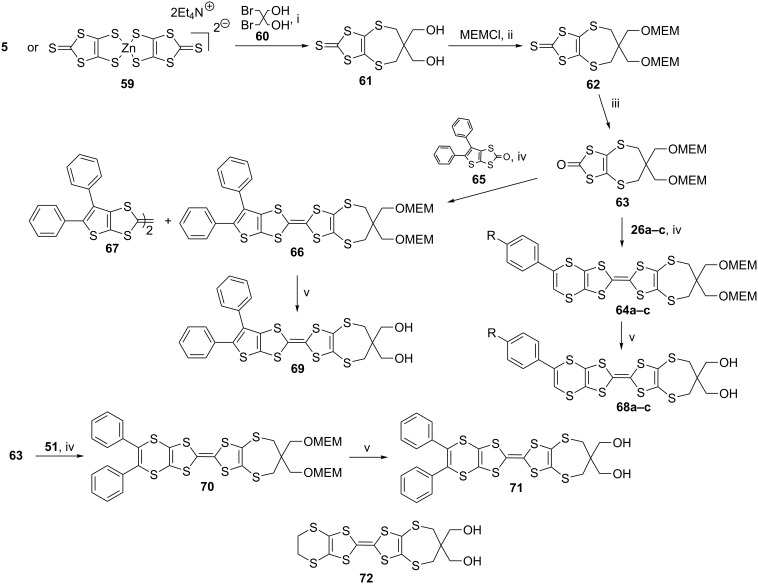
Reaction conditions (i) EtOH, reflux, overnight; (ii) diisopropylethylamine in CH_2_Cl_2_, room temperature, overnight; (iii) (AcO)_2_Hg/AcOH, CHCl_3_, rt, 3h; iv) (EtO)_3_P, 110 °C, N_2_, 2 h; (v) sample in THF at 0 ^o^C, add 20% HCl, then rt, overnight.

Analogues of ET, having dithiin and thiophene rings along with hydroxy groups were synthesized to provide the possibility of intramolecular hydrogen bonding through the hydroxy groups [57]. The half ET analogue **61** was obtained from the reactions of either the dianion **5** or the zinc-complex **59** with 2-bis(bromomethyl)propane-1,3-diol (**60**, Scheme 10). As the hydroxy groups could lead to side products during the coupling reaction, performed using triethyl phosphite, and the reaction for conversion of the thione group to a keto group with mercury acetate and acetic acid, they were protected by reaction with methoxyethoxymethylchloride (CH_3_OCH_2_CH_2_OCH_2_Cl, MEMCl) to obtain **62**. The thione group of this compound, was then converted into a keto group to give **63**. Its reaction with **26a–c** in triethyl phosphite led to the formation of cross-coupled product **64a–c**, along with the self coupled one. Following the same procedure, the half ET analogue **65**, possessing a fused diphenylthiophene ring was coupled with **63** to produce **66**, along with self coupled products like **67**. Removal of the MEM groups of both **64** and **66** in dilute HCl/THF mixture resulted in the formation of the target analogues **68a–c** and **69**, having two hydroxy groups. Coupling of the dithiinone **51** with **63** gave **70** and its hydrolysis yielded the ET analogue **71**, possessing diphenyldithiin and two hydroxy groups. Following the same strategy, an ET analogue **72**, having half ET and two hydroxy groups was synthesized to compare the oxidation and reduction potentials of the analogues. The yields of the resultant products are given in Table 4.

**Table 4:** Yields of the products **64**, **65**, **68**–**71**.

Product	Yield (%)	Diol	Yield (%)

**70**	30	**71**	75
**65**	30	**69**	75
**64a**	27	**68a**	43
**64b**	29	**68b**	35
**64c**	40	**68c**	34

The oxidation and reduction properties of the diphenyl analogues **56**, **69** and **71** and monophenyl analogues **68a–c, 31a** and **29a** were investigated and compared by cyclic voltammetry (CV) (Table 5) with ET **3** and its fully unsaturated tetraphenyl analogue **52**. The CV measurement of the donors was performed in 0.1 M NaClO_4_/ACN with a scan rate of 100 mVS^−1^ at room temperature, using Pt working and Ag/Ag^+^ reference electrodes. The spectroelectrochemical studies were carried out in CH_2_Cl_2_ containing 0.1 M TBABF_4_ at room temperature.

**Table 5:** Redox potential of ET **3** and its analogues, ACN solution of 0.1 M NaClO_4_.

Sample	Oxidation potential (V)		

	*E*^1^_OX_ (V)	*E*^2^_OX_ (V)	Δ*E* (V)

**52**	0.72	1.03	0.31
**70**	0.44	0.70	0.26
**66**	0.60	0.84	0.24
**71**	0.41	0.63	0.22
**69**	0.57	0.80	0.23
**56**	0.36	0.59	0.23
**68a**	0.49	0.74	0.25
**68b**	0.50	0.76	0.26
**68c**	0.42	0.70	0.28
**67**	0.72	1.06	0.34
**31a**	0.49	0.63	0.14
**72**	0.42	0.70	0.28
**29a**	0.66	0.96	0.30
**3**ET	0.46	0.71	0.25

The CV studies indicated that while the fully unsaturated **52** and diphenylthiophenedimethylthio **67** had the highest oxidation potentials, diphenyldithiindimethylthio **56** displayed the lowest oxidation potential and combination of dithiin and diol groups led to oxidation potentials comparable with ET **3**.

**Scheme 11:**
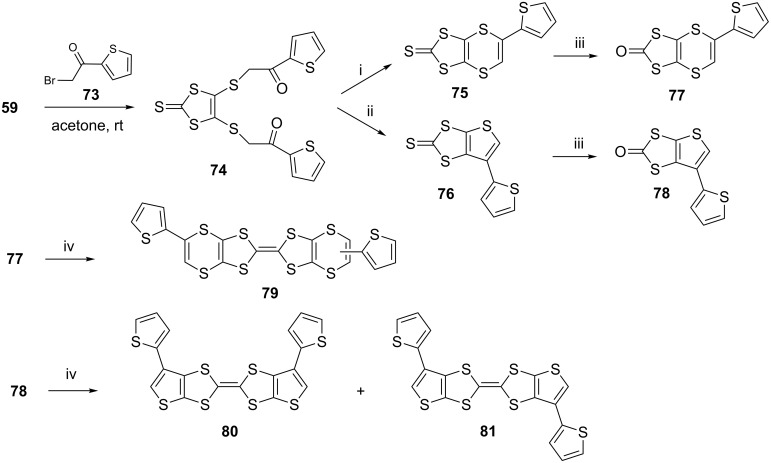
Reaction and conditions (i) P_4_S_10_, NaHCO_3_, toluene, reflux, 3 h; (ii) P_4_S_10_, *p*-TSA, toluene, reflux, 3 h; (iii) (AcO)_2_Hg/AcOH, CHCl_3_, 3 h, rt; (iv) (EtO)_3_P, 110 °C, N_2_, 2 h.

BEDT-TTF analogues possessing thiophene substituted 1,4-dithiin and thiophene rings were reported in 2013 [58]. Their syntheses began with our standard synthesis of a 1,8-diketone **74** having a thiophene in place of a benzenoid aromatic group (Scheme 11). Reaction of the zinc-complex **59** with four mol equivalents of α-bromoketone **72** gave the diketone **74** in 80% yield, subsequent ring closure of which with P_4_S_10_ in acidic and basic conditions produced 1,4-dithiin **75** (75%) and thiophene **76** (57%) rings, respectively. They were then converted into their corresponding oxo forms **77** (65%) and **78** (77%), respectively, with mercury acetate and subjected to the coupling reaction with triethyl phosphite to produce the ET analogues having 1,4-dithiin rings **79** (80%) and thiophene rings **80** and **81** (75%) as inseparable isomers.

Unfortunately, all attempts to electropolymerize the analogues failed. Computational studies indicated that the α-carbons of the peripheral thiophenes, where the polymerization was expected to take place, did not exhibit enough spin density.

## Conclusion

Bis(ethylenedithio)tetrathiafulvalene (BEDT-TTF, ET) is a unique molecule which has been successfully used as an electronic material that challenges the creativity and inventiveness of chemists in areas such as organic chemistry, materials chemistry, supramolecular chemistry and polymer chemistry. 1,8-Diketones have been demonstrated to be versatile starting materials for the synthesis of various challenging analogues of ET, possessing dithiin and thiophene moieties. This chemistry not only led to the production of the target materials, having interesting electronic properties, but also illustrated challenging synthetic heterocyclic chemistry.
